# Successful Immunotherapy for Pancreatic Cancer in a Patient With TSC2 and SMAD4 Mutations: A Case Report

**DOI:** 10.3389/fimmu.2021.785400

**Published:** 2021-11-22

**Authors:** Yanghui Ye, Song Zheng

**Affiliations:** ^1^ The Fourth School of Clinical Medicine, Zhejiang Chinese Medical University, Hangzhou, China; ^2^ Department of Oncology, Affiliated Hangzhou First People’s Hospital, Zhejiang University School of Medicine, Hangzhou, China; ^3^ Department of Oncology, Affiliated Hangzhou Cancer Hospital, Zhejiang University School of Medicine, Hangzhou, China; ^4^ Laboratory of Clinical Cancer Pharmacology and Toxicology Research of Zhejiang Province, Hangzhou, China

**Keywords:** pancreatic cancer, immunotherapy, chemotherapy, next generation sequencing (NGS), SMAD4, TSC2

## Abstract

**Background:**

Pancreatic cancer has a poor prognosis, and it is traditionally treated with chemotherapy. Fortunately, immunotherapy has rapidly changed the landscape of solid tumor treatment, and improving the survival of cancer patients. However, pancreatic cancer is non-immunogenic, and single agent immunotherapies are unfavorable to its prognosis.

**Case Presentation:**

Here, we report a case of stage IV pancreatic cancer in a patient with TSC2 and SMAD4 mutations treated with immunotherapy when the disease progressed after multi-line chemotherapy. Next generation sequencing (NGS) confirmed the presence of TSC2 and SMAD4 mutations and microsatellite stability (MSS). When the disease progressed after chemotherapy, a combination strategy was devised consisting of chemotherapy (S-1) and sintilimab. The patient had a partial response to therapy with this regimen, the lesions were significantly reduced and nearly disappeared. In metastatic pancreatic cancer, responses of this magnitude are rarely seen.

**Conclusions:**

This outcome reveals that this combination can be effective in treating metastatic pancreatic cancer, especially in pancreatic cancer patients with SMAD4 and TSC2 mutations. This may help increase the use of this therapy in large-scale clinical research.

## Introduction

Pancreatic cancer has a high incidence and mortality, and its special structure can protect pancreatic cancer cells from chemotherapeutic agents (1, 2). However, pancreatic cancer is non-immunogenic and single agent immunotherapies are unfavorable to the prognosis of patients. Several clinical trials showed that single agent immunotherapies are ineffective for the treatment of advanced pancreatic cancer (3–5). Moreover, there was a clinical trial confirmed that objective response rate (ORR) was 0 for patients receiving single agent immunotherapies (5).

However, the effect of combination therapy is also not optimistic. As for chemotherapy plus immunotherapy, some clinical trials confirmed that the safety profile of combination therapy at standard doses in advanced pancreatic cancer was manageable (6, 7), but there was no significant improvement in progression-free survival (PFS) and overall survival (OS) (6, 8).

Though, the pancreatic cancer has little response rate to immunotherapy, it may be effective for specific patient. For example, several gene mutations can improve the effective of immunotherapy (9), including high levels of microsatellite instability (MSI-H), POLE, POLD1, et al.

## Case Presentation

**Figure 1:**
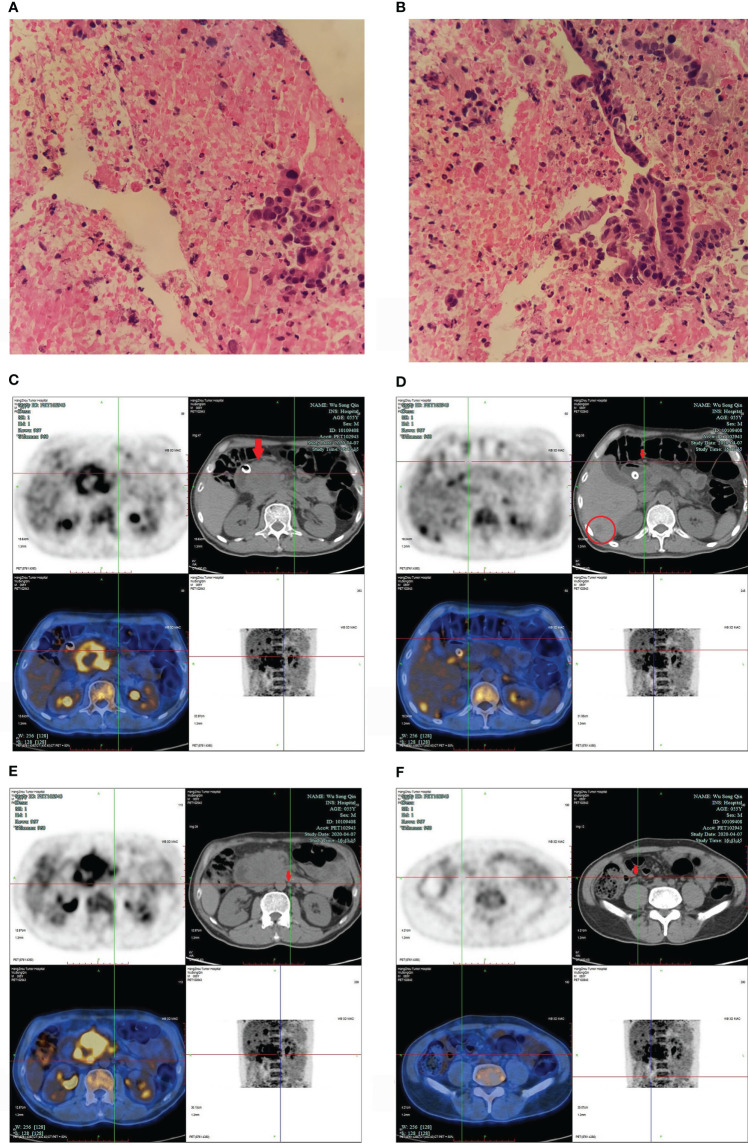
**(A, B)** pancreatic ductal adenocarcinoma. **(A)** 200X, **(B)** 400X. The pancreatic head puncture smear showed significant atypical epithelial mass, irregular nuclei, which was consistent with the changes of adenocarcinoma. **(C–F)** PET/CT images in April 2020. **(C)** showed a 6.0cm *6.0 cm mass in pancreatic head (arrow); **(D)** confirmed enlarged lymph nodes in pancreatic head (arrow), and low-density shadows in liver (within cycle); panels **(E, F)** revealed enlarged multiple lymph nodes in different sizes in retroperitoneum (arrow).

We present the case of a 56-year-old Chinese man who had been smoking and drinking for decades. He was hospitalized for six months with abdominal pain. In September 2019, magnetic resonance imaging (MRI) of the upper abdomen identified a pancreatic head mass, multiple retroperitoneal enlarged lymph nodes, and abnormal enhancement near the inferior vena cava in right lobe of the liver. Ultrasound guided biopsy of the pancreas was performed. Pathology diagnosis was pancreatic ductal adenocarcinoma (stage IV) (**Figures 1A, B**). Next generation sequencing (NGS) confirmed the tumor was microsatellite stability (MSS), and a total of 6 gene mutations, TSC2, CREBBP, HIST1H3I, MAP2K4, SMAD4, and STK11 were detected. Additionally, frame shift mutation occurred in exon 17 of TSC2 gene (32.13%); missense mutation occurred in exon 9 (7.47%) and nonsense mutation occurred in exon 5 (9.49%) of SMAD4 gene. The NGS confirmed that there were no targeted drug-related gene mutations and the tumor mutational burden (TMB) was 7.1 mut/Mb.

In October 2019, the patient’s primary oncologist started him on AG (gemcitabine and nab-paclitaxel). The gemcitabine (1000mg/m² over 30 minutes, weekly for 2 weeks, every 21 days) and nab-paclitaxel (125mg/m², weekly for 2 weeks, every 21 days) were administered intravenously. However, in the first three cycles and the fifth cycle of chemotherapy, for several reasons (such as COVID-19), he only received chemotherapeutic agents (AG) on the first day. In March 2020, the repeated MRI showed progressive disease. In late March 2020, the patient developed back pain and underwent endoscopic retrograde cholangiopancreatography (ERCP) and endoscopic metal biliary endoprosthesis (EMBE) due to obstructive jaundice. In April 2020, positron emission tomography/computed tomography (PET/CT) revealed a 6.0 cm × 6.0 cm mass in pancreatic head, multiple enlarged lymph nodes in different sizes were found in pancreatic head and retroperitoneum, and multiple low-density shadows in the liver (**Figures 1C–F**).

**Figure 2:**
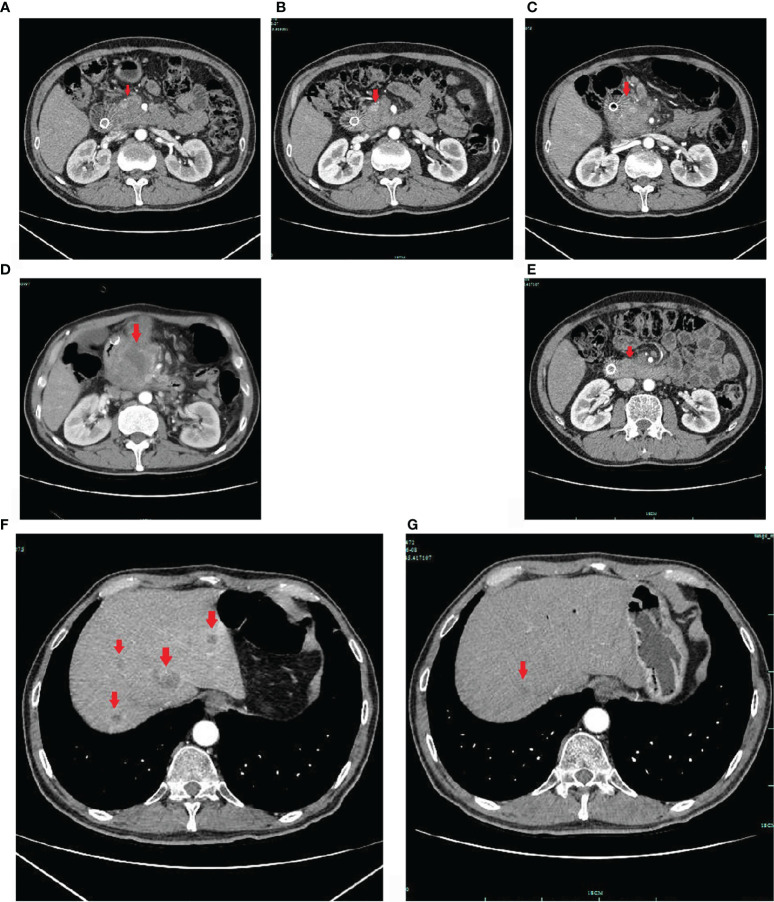
Enhanced CT images. The pancreatic mass indicated by the arrow on upper-abdomen enhanced CT took in June 2020 **(A)**, in August 2020 **(B)**, in October 2020 **(C)** and in November 2020 **(D)**. The pancreatic cancer was responded to chemotherapy initially, but the disease was still progressed at later time. After sintilimab combined with S-1 treatment, the pancreatic lesion was significantly reduced in, and nearly disappeared in June 2021 **(E)**. The hepatic metastases indicated by the arrows on upper-abdomen enhanced CT took in October 2020 **(F)**, and in June 2021 **(G)**. The hepatic metastasis was significantly reduced **(G)**.

Based on these findings, the patient was treated with FOLFIRINOX (oxaliplatin, Irinotecan, calcium folinate and 5-Fluorouracil). However, considering that the patient had just undergone surgery, the patient was asked by his doctor to eat S-1 for 14 days (from April 2, 2020 to April 16, 2020) before he received FOLFIRINOX treatment on April 25, 2020. In June 2020, after 4 cycles of chemotherapy, upper-abdomen enhanced CT revealed that the size of pancreatic head lesion was significantly decreased to 2.3 cm × 2.5 cm (**Figure 2A**), more that 50% decrease, and multiple low-density shadows in the liver. The patient was repeated CT about every two months (**Figures 2B, C**). Until October 2020, the CT only revealed a mass in the pancreatic head, and the lesion was slightly low density with peripheral lymph node metastasis.

In October 2020, the patient underwent one cycle of Olaparib by himself, although he did not have the targeted drug-related gene mutation. On October 16, his abdomen enhanced CT scan revealed multiple liver metastases (**Figure 2F**), and the largest measured 2.3 cm. We changed the chemotherapy regimen to AG again (gemcitabine and nab-paclitaxel). In November 2020, the abdomen enhanced CT scan revealed progressive disease (**Figure 2D**), and the size of the pancreatic head lesion was 6.4 cm.

The patient has undergone multi-line chemotherapy, chemotherapy alone could not inhibit the progression of the disease, and there was no standard treatment after two or more lines of systemic chemotherapy. In addition, his Eastern Cooperative Oncology Group Performance Status (ECOG-PS) was 2, he suffered from severe cancer pain all over the body (OxyContin 30mg, every 12 hours) and weight loss, the aspartate transaminase and alanine transaminase were at normal level, and alkaline phosphatase increased slightly (not higher than 2 times the normal value). Thus, we decided to use chemotherapy (S-1) combined with immunotherapy (sintilimab). Considering the economic status of patient, we decided to choose sintilimab (more economical). The sintilimab is a fully human IgG4 monoclonal antibody; it can bind to PD-1, block the interaction of PD-1 with its ligands, and help recover the anti-tumor response of T-cells, and it has been approved to treat relapsed or refractory classical Hodgkin lymphoma, advanced non-small cell lung cancer and metastatic hepatocellular carcinoma. The anti-tumor effect of sintilimab is similar to that of other anti-PD-1/PD-L1 antibodies (10). Sintilimab was administered intravenously (200mg, every 3 weeks); and S-1was orally (40mg, twice a day orally for 2 weeks, every 21 days). The patient started to receive S-1 on November 24, 2020, and sintilimab on November 27, 2020.

**Figure 3:**
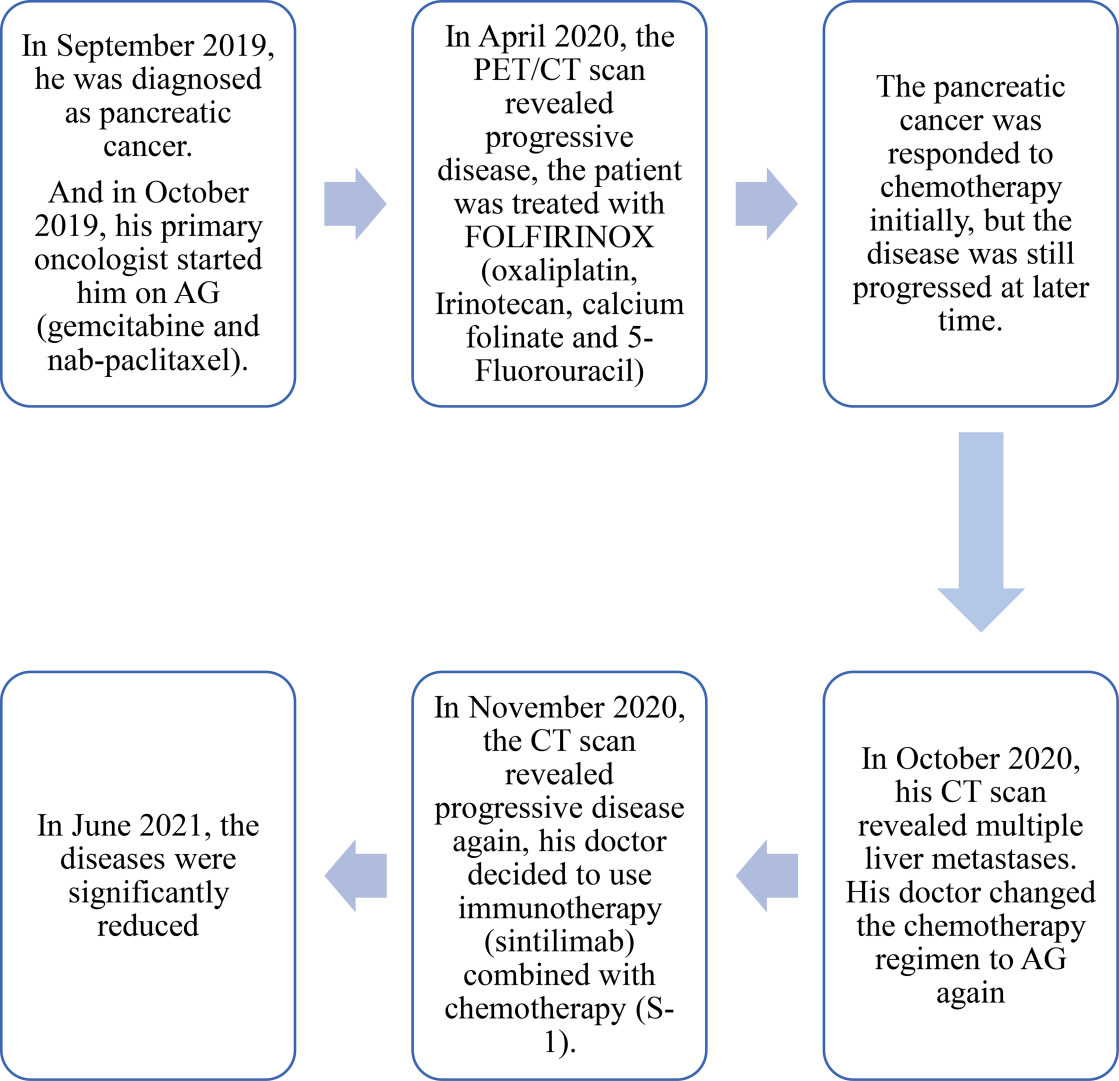
Timeline of the treament. The patient was diagnosed with pancreatic cancer in September 2019, and received chemotherapy (AG) in October 2019. Before receiving immunotherapy in November 2020, the patient had undergone multi-line chemotherapy (including AG, FOLFIRINOX, AG). And until August 2021 (the latest follow-up), the patient was still receiving immunotherapy combined with chemotherapy.

The patient did not come to our hospital again until June 2021 (**Figure 3**). He said he was treated at a local hospital during these seven months, and received a total of 10 cycles of sintilimab combined with S-1. The performance status was better than before (his PS improved to ECOG-PS 1); his severe cancer pain was well controlled (OxyContin 10mg, every 12 hours). The abdomen enhanced CT scan showed that the intrahepatic metastases (**Figure 2G**) and pancreatic head lesion (**Figure 2E**) were significantly reduced (the size of the pancreatic head lesion was 1 cm). By standard Response Evaluation Criteria in Solid Tumors (RECIST) criteria, version 1.1, the patient had a partial response (more than 80% decrease) to therapy with this regimen. Moreover, throughout his treatment, the CA 19-9 level was always normal.

At the latest follow-up (August 2021), the patient still had a partial response to this regimen (S-1 combined with sintilimab), and the duration of partial response was 8 months.

## Discussion

Chemotherapy has been the mainstay of treatment for many malignant tumors, including pancreatic cancer. Due to the lack of effective screening tools, most patients lose the opportunity to undergo surgery, which makes chemotherapy the standard treatment. However, chemotherapy has a poor therapeutic effect on pancreatic cancer due to its aggressive nature. Pancreatic cancer is composed of malignant cells and desmoplastic stroma (1). Desmoplastic stoma serves as a physical barrier that protects pancreatic cancer cells and prevent the effective delivery of chemotherapeutic agents (2).

In this case, the chemotherapy was effective for this patient at first. Additionally, after the disease progressed, the immunotherapy combined with chemotherapy had a significant effect. In the past 5 years, immunotherapy has rapidly changed the landscape of solid tumor treatment. What is more, tumor testing can help patients to get a better treatment. The results of a tumor testing making a patient eligible for treatment with immunotherapy, whose disease (advanced pancreatic cancer) progressed after neoadjuvant chemotherapy and adjuvant chemotherapy (11). Recent studies have identified several positive predictive markers for immune checkpoint inhibitors (ICIs), such as high levels of MSI-H, PD-L1 overexpression, high TMB, and gene mutations (9).

TSC2 is a tumor suppressor gene; it negatively regulates the cellular signaling networks that control cellular growth and proliferation (12). The TSC2 protein forms a complex and functions as a tumor suppressor by inhibiting mTORC1 kinase (13). It has been shown that, in TSC2-deficient tumors, the single-agent PD-1 or CTLA-4 blockade, or a combination of them, can inhibit the growth of tumors (14). Additionally, the combination of PD-1 and CTLA-4 antibody treatment or single-agent treatment can increase CD8+ T-cell infiltration in TSC2-deficient human tumors (14), and the level of infiltration is correlated with the degree of response to therapy.

Transforming growth factor beta (TGF-β) is an immune regulator; it can suppress the immune response *via* many different mechanisms (15). Moreover, it can inhibit tumor growth at the early stages of disease and promote tumor development at the later stages (16). However, the tumor-suppressive role of TGF-β is only effective when the TGF-β signaling pathway is not defective (17). SMAD4 serves as the central mediator of the TGF-β signaling pathway (18), and it is the only common mediator. The TGF-β/SMAD4 signaling pathway plays a tumor suppressive role in early stages of disease, mainly by inducing cell cycle arrest and apoptosis. TGF-β can stimulate regulatory T-cells, which inhibit the function of other lymphocytes (19). PD-1 is highly expressed on tumor infiltrating lymphocytes; it has been shown that human PD-1 expression may under direct transcriptional control by TGF-β, and TGF-β can enhance the expression of PD-1, suppressing anti-tumor immunity (20). TGF-β inhibits CD8+ T-cell effector function through TGF-β signaling pathway (21). Pancreatic cancer cells have lost their tumor-suppressive roles, but they possess tumor-promoting effects induced by increased TGF-β (22). In a tumor microenvironment, TGF-β expression is very high.

In pancreatic cancer, alterations of TGF-β signaling occur through the mutation of the genes involved in the pathway (including SMAD4); this activity is present in 47% of pancreatic cancer patients (23). The loss of SMAD4 will abrogate the canonical TGF-β/SMAD4 signaling pathway (24), and it may make pancreatic cancer more aggressive (25). It has been shown that SMAD4-deleted pancreatic ductal adenocarcinoma cells are sensitive to agents modulating the cell cycle (26). The loss of SMAD4 counteracted TGF-β-induced cell cycle arrest and apoptosis (27). Furthermore, it has been reported that the loss of SMAD4 expression is significantly associated with better survival after resection (28). The inhibition of TGF-β has been reported to have a variety of antitumor effects (29). A TGF-β blockade can reverse the suppressive effects of apoptotic cells on inflammation and adaptive immunity (30). In T-cell excluded mouse models, immune checkpoint-resistant MSS colorectal cancers and liver tumors were rendered susceptible to anti-PD-1/PD-L1 therapy with a TGF-β blockade (31).

Blockade of immune checkpoints by anti-CTLA-4 or anti-PD-1/anti-PD-L1 agents leads to T-cell activation, and it provides an effective approach for tumor immunotherapy (32). And the high PD-L1 expression may have a better clinical benefit. There was a case report showed that blocking the PD-L1 pathway combined with chemotherapy was effective for pancreatic squamous cell carcinoma patients with high PD-L1 expression (33).

Pancreatic cancer is intrinsically non-immunogenic (34). Single agent immunotherapies are unlikely to be successful in treating this type of cancer (35), but immunotherapy combined with chemotherapy has a synergistic effect (36). Chemotherapeutic agents could promote the release of tumor antigens from the cancer cells and reactivate an anti-cancer immune response to suppress tumor growth (37). Besides, according to several ongoing clinical trials, there are other regimens of combination therapy for the treatment of pancreatic cancer, such as BL-8040 (chemokine receptor type 4 inhibitors) and pembrolizumab combined with chemotherapy (NCT02826486), olaparib plus pembrolizumab (NCT04666740 and NCT04548752), olaparib or selumetinib plus durvalumab (NCT04348045). Additionally, the COMBAT/KEYNOTE-202 Trial (NCT02826486) revealed that the ORR was 21.1%, and the triple combination of BL-8040, pembrolizumab, and chemotherapy was safe and well tolerated, but no significant improvement in PFS and OS (38).

Other ICIs can also improve the effect of therapy. TMB is the total number of mutations per coding area of a tumor gene, which can increase the sensitivity to immunotherapy (39). Generally, we defined TMB ≥ 20 mutations/Mb as high TMB, TMB ≤ 10 mutations/Mb as low TMB. Patients with a high TMB also have a better prognosis with immunotherapy. For example, a higher TMB was associated with better response in non-small cell lung cancer patients receiving pembrolizumab (40). A higher TMB had a clinical benefit in malignant melanoma patients receiving either ipilimumab or tremelimumab (41). Additionally, a case report revealed that combined antiangiogenic therapy and immunotherapy is effective for pancreatic cancer with high TMB (42). However, patients with pancreatic cancer generally have a low TMB in comparison to patients with other malignancies (43). Moreover, pancreatic cancer is a tumor with low immunogenicity, which is attributed to low TMB (36).

## Conclusion

In this case, the effect of immunotherapy combined with chemotherapy seems to be very effective. We also established a hypothesis that the SMAD4 and TSC2 mutations improved the efficacy of immunotherapy, prolonging the survival of patients. However, very few studies have investigated the relationship between SMAD4 mutation and immunotherapy in pancreatic cancer. Thus, more studies and clinical trials are needed.

## Data Availability Statement

The original contributions presented in the study are included in the article/supplementary material. Further inquiries can be directed to the corresponding author.

## Author Contributions

SZ is the guarantor. YY wrote the manuscript. All authors read, provided feedback, and approved the final version.

## Funding

The National Natural Science Foundation of China (81372660), Zhejiang Province Public Welfare Technology Application Research Project (2017C33200), and Laboratory of Clinical Cancer Pharmacology and Toxicology Research of Zhejiang Province.

## Conflict of Interest

The authors declare that the research was conducted in the absence of any commercial or financial relationships that could be construed as a potential conflict of interest.

## Publisher’s Note

All claims expressed in this article are solely those of the authors and do not necessarily represent those of their affiliated organizations, or those of the publisher, the editors and the reviewers. Any product that may be evaluated in this article, or claim that may be made by its manufacturer, is not guaranteed or endorsed by the publisher.

## Abbreviations

MRI, magnetic resonance imaging; PET, positron emission tomography; CT, computed tomography; NGS, next generation sequencing; TGF-β, transforming growth factor beta; TMB, tumor mutational burden; MSS, microsatellite stable; MSI, microsatellite instability; MSI-H, high levels of microsatellite instability; ICI, immune checkpoint inhibitor; ORR, objective response rate; PFS, progression-free survival; OS, overall survival.
